# Implementation of a reference standard and proficiency testing programme by the World Wide Antimalarial Resistance Network (WWARN)

**DOI:** 10.1186/1475-2875-9-375

**Published:** 2010-12-25

**Authors:** Chris Lourens, William M Watkins, Karen I Barnes, Carol H Sibley, Philippe J Guerin, Nicholas J White, Niklas Lindegardh

**Affiliations:** 1 Worldwide Antimalarial Resistance Network (WWARN), Oxford, UK; 2 Mahidol Oxford Research Unit, Faculty of Tropical Medicine, Mahidol University, Bangkok Thailand; 3 Centre for Tropical Medicine, Nuffield Department of Medicine, University of Oxford, Oxford, UK; 4 Division of Clinical Pharmacology, Department of Medicine, University of Cape Town, Cape Town, South Africa; 5 Department of Genome Sciences, University of Washington, Seattle, WA, USA

## Abstract

**Background:**

The Worldwide Antimalarial Resistance Network (WWARN) is a global collaboration to support the objective that anyone affected by malaria receives effective and safe drug treatment. The Pharmacology module aims to inform optimal anti-malarial drug selection. There is an urgent need to define the drug exposure - effect relationship for most anti-malarial drugs. Few anti-malarials have had their therapeutic blood concentration levels defined. One of the main challenges in assessing safety and efficacy data in relation to drug concentrations is the comparability of data generated from different laboratories. To explain differences in anti-malarial pharmacokinetics in studies with different measurement laboratories it is necessary to confirm the accuracy of the assay methods. This requires the establishment of an external quality assurance process to assure results that can be compared. This paper describes this process.

**Methods:**

The pharmacology module of WWARN has established a quality assurance/quality control (QA/QC) programme consisting of two separate components:

1. A proficiency testing programme where blank human plasma spiked with certified reference material (CRM) in different concentrations is sent out to participating bioanalytical laboratories.

2. A certified reference standard programme where accurately weighed amounts of certified anti-malarial reference standards, metabolites, and internal standards are sent to participating bioanalytical and in vitro laboratories.

**Conclusion:**

The proficiency testing programme is designed as a cooperative effort to help participating laboratories assess their ability to carry out drug analysis, resolve any potential problem areas and to improve their results - and, in so doing, to improve the quality of anti-malarial pharmacokinetic data published and shared with WWARN.

By utilizing the same source of standards for all laboratories, it is possible to minimize bias arising from poor quality reference standards. By providing anti-malarial drug standards from a central point, it is possible to lower the cost of these standards.

## Background

The Worldwide Antimalarial Resistance Network (WWARN) is a global collaboration, established in 2009, with the support of the Bill and Melinda Gates Foundation. The aim is to support the objective that anyone affected by malaria receives effective and safe drug treatment. Working closely with WHO, WWARN has the goal of providing urgently needed, comprehensive, timely and quality assured information to track the emergence and spread of anti-malarial drug resistance so that global efforts to control the disease are founded on reliable information. WWARN validates and provides tools to obtain this information and shares data from a continuously updated database. The Network supports and enhances relevant research capacity in malaria-endemic countries and is coordinated through regional centres established in malaria-endemic areas.

The Asia Regional Centre is the first such coordinating centre, operating out of Mahidol University in Bangkok, Thailand. WWARN is composed of six complementary modules [1] Clinical, Pharmacology, In vitro, Molecular, Informatics and Anti-malarial Quality. Achieving adequate anti-malarial drug concentrations in the blood is essential for reliable cure of malaria. The Pharmacology module aims to inform optimal anti-malarial drug selection and dosing by characterizing individual and population blood concentration profiles, thereby assessing the therapeutic ratio in different patient groups, estimating selection pressures and differentiating resistance from under-dosing. This review outlines the approaches taken and methods used by the WWARN Pharmacology module.

### Pharmacology module

Therapeutic failure is not always an indication of resistance to the malaria parasite. It may be the result of poor drug quality, inadequate dosing, vomiting, poor absorption, or reduced drug exposure because of increases in the apparent volume of distribution or clearance. Moreover, it is essential that anti-malarial drug concentration profiles are characterized in all key target populations so that dosage regimens can be optimized. The choice of the optimal dose is dependent on many, often interrelated factors such as tablet size, acceptability, toxicity as well as the pharmacokinetic properties of the drug(s) used (which may vary with e.g. age, body mass, disease severity, coadministered food, and pregnancy). There is always a trade-off between simplicity and accuracy of the mg/kg dose. Low dosage increases the risk of treatment failure while high dosage may increase side effects. As current dose recommendations were often developed for acute uncomplicated malaria in adults, vulnerable populations (e.g. infants, malnourished children or pregnant women) may receive inappropriate dosage of the currently recommended anti-malarials [2-5].

There is an urgent need to define the drug exposure - effect (i.e. the pharmacokinetic-pharmacodynamic; PK-PD) relationship for most anti-malarial drugs. Few anti-malarials have had their therapeutic blood concentration levels defined [6-9]. Studies on artemisinin combinations have shown that a single time point (i.e. the day 7 concentration of the slowly eliminated partner drug) is a valuable determinant of total drug exposure and consequently treatment outcome [6,7,9].

One of the main challenges in assessing safety and efficacy data in relation to drug concentrations in various patient populations is the comparability of data generated from different laboratories. The same issues bedevils comparison of in vitro susceptibility assessments [10]. To explain differences in anti-malarial pharmacokinetics in studies with different measurement laboratories (or assays performed at different times) it is necessary to confirm the accuracy of the assay methods. This requires the establishment of an external quality assurance process to assure results that can be compared [11]. This paper describes how this process was set up. The results from different laboratories can now be compared through a proficiency testing programme [12]. Inter-laboratory biases can be minimized through the use of reliable reference standards, implementation of quality systems, and external proficiency testing.

The pharmacology module of WWARN has established a quality assurance/quality control (QA/QC) programme consisting of two separate components:

1. A proficiency testing programme where blank human plasma spiked with certified reference material (CRM) in different concentrations is sent out to participating bioanalytical laboratories. The quality control samples will help laboratories in the tracking of potential problem areas. The QA/QC unit will also provide assistance and advice to help laboratories solve quality or methodological issues that might arise during testing.

2. A certified reference standard programme where accurately weighed amounts of certified anti-malarial reference standards, metabolites, and internal standards are sent to participating bioanalytical and in vitro laboratories.

### Rationale

The pharmacology module of WWARN is dedicated to provide the necessary tools to ensure that pharmacology data generated around the globe is reliable and of a high standard. Informing optimal anti-malarial selection and dosing requires the global cooperation of researchers active in anti-malarial pharmacology to define adequately therapeutic drug concentrations and facilitate a better understanding of the patient, dosing and disease factors that change anti-malarial exposure sufficiently to compromise the anti-malarial efficacy (or safety). From the above it is evident that a well controlled pharmacology QA/QC programme will not only benefit the quality of data to be uploaded to the WWARN database, but also patients, contributing to the control, and eventual elimination, of malaria.

### Definitions

#### Quality assurance (QA)

QA aims at improving and stabilizing processes to minimize or avoid issues that lead to unreliable results.

#### Quality control (QC)

QC also known as external quality assessment (EQA) or proficiency testing (PT) emphasizes testing and blocks the release of unreliable results.

### Reference material programme

**Figure 1:**
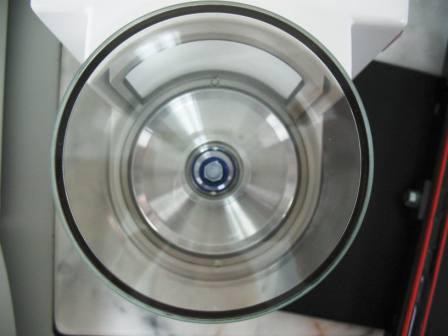
**Micro weighing boat on balance**.

**Figure 2:**
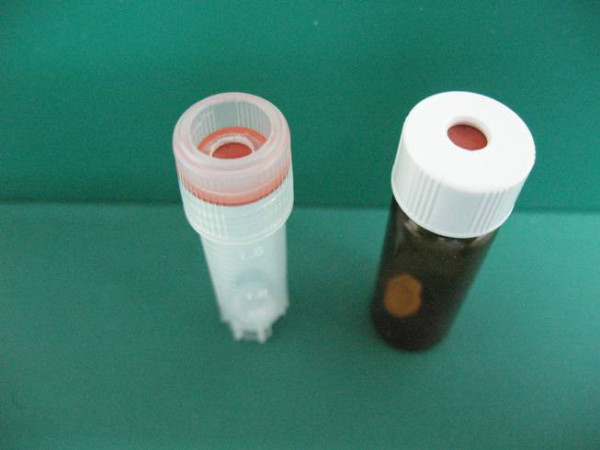
**Micro weighing boat in cryovial**.

The reference material programme is based in the Pharmacology Department of the Mahidol-Oxford Research Unit, Faculty of Tropical Medicine, Mahidol University, Bangkok but operates as an independent entity. By providing anti-malarial drug standards from a central point, the total cost of these standards can be lowered substantially. Initially, laboratories may freely participate in the QA/QC programme and receive standards and proficiency testing samples without charge. In the future, WWARN will have to introduce a cost-recovery system to ensure the programme is sustainable, whilst maintaining wide access to the services. Participants will be notified of any intended changes in advance. The QA/QC unit acquires all anti-malarial drug standards, metabolite standards and internal standards from an independent contract research and development organization that specializes in the synthesis of stable isotope-labelled compounds, metabolites and pharmaceutical related substances. All reference materials and standards are accompanied by certificates of analysis (CoA). Re-certification of each material is done 1 month prior to expiry date. Reference standards are stored at the QA/QC unit under optimal conditions and humidity, temperature, and stability data are monitored continuously to maximize usage of the materials. By utilizing the same source of standards and same weighing procedures for all laboratories, it is possible to minimize bias arising from poor quality standards and weighing inaccuracies. The QA/QC unit sends out small, accurately weighed samples of anti-malarial reference standards, metabolites and internal standards to research groups that are registered with the programme. Common standards and metabolites will be supplied in 20 mg quantities while rare metabolites and internal standards will be accurately weighed in amounts of 0.4000 - 2.000 mg into inert disposable micro weighing boats on a sensitive ultra micro balance (Figures 1 and 2). Custom made inert disposable micro weighing boats will be available to the laboratories upon request.

**Figure 3:**
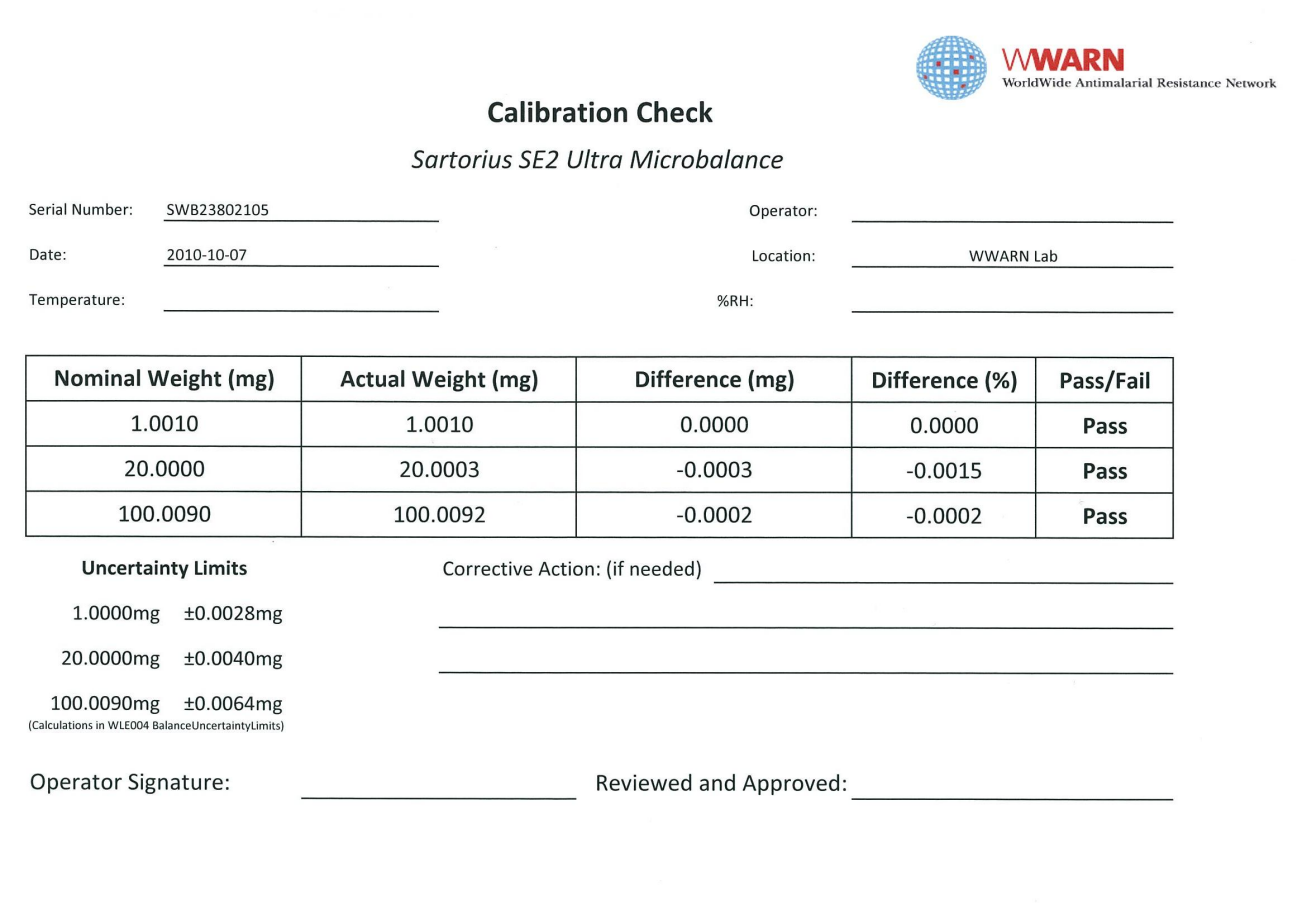
**Example of Calibration Check certificate**.

The balance has an uncertainty at the 1 mg level of ±0.0013 mg. The balance is calibrated annually with traceable weights by an accredited company. The calibration certificate is kept on file and a copy can be obtained upon request. As an example, if the amount of 0.4000 mg is weighed it will introduce an error of less than ±0.33% of the final result. The overall procedure used is based on Good Weighing Practice (GWP) principles and is described in internal standard operating procedures. Various tests were done under different conditions to obtain the optimum conditions prior to commencement of weighing standards. Tests were done for accuracy, precision and stability of the balance by repeated weighing Class E2 weights and then calculating averages and standard deviations over varying times. As an example, for six series of sixty readings each (every five seconds for five minutes) a mean weight of 1.0011 mg (sd 0.0002) was obtained from a weight certified to have a nominal weight of 1.0010 mg. The balance is housed in a plexiglass cabinet on a stable vibration-free platform and is always in a standby state. The room is temperature and humidity controlled. Local power supply is complimented by an uninterruptable power supply (UPS) and is sufficiently stable. The balance is turned on and left to stabilize for four hours before use. Three different weights are checked and the results recorded on a log sheet (see Figure 3). This log sheet indicates a 'Pass' or 'Fail' status for each weight using calculated uncertainty limits for both the weights and the balance. Balance check weights are Class E2. These weights are calibrated quarterly against traceable weights by an independent accredited company. The weight calibration check is carried out before each series of weighings. A copy of the log sheet is provided with every mailing (Figure 3). Common standards and metabolites supplied in 20 mg quantities will need to be accurately weighed out by the receiving laboratory before use. Rare metabolites and internal standards are weighed out accurately by the QA/QC unit and can be used (dissolved) as they arrive. The precise weight of each rare metabolite or internal standard is recorded automatically and electronically before the weighing boat is transferred to a 2 ml cryovial. A set of two cryovials for each standard is sent to requesting laboratories where an appropriate amount of solution will be added directly to the tube to produce Reference Stock Solutions (RSS) ranging between 0.4-2.0 mg/ml. It is recommended that two vials (i.e. one set) are reconstituted each time an RSS needs to be prepared. The two vials (RSS1 and RSS2) should be analysed using appropriate methods at the site and the result compared. An alternative approach is to prepare standards using RSS1 and QC samples using RSS2, analyse them and compare the results. The deviation from the stated weight should be <|method imprecision| + 2%. These RSS have a shelf-life of up to 1 month when stored at 4°C. Many RSS can also be successfully stored as aliquots at -80°C with an increased shelf-life.

### Proficiency testing (PT) programme

Day-to-day running of the PT programme is the responsibility of the WWARN QA/QC unit while the long-term strategic planning is decided together with an international programme advisory committee comprising an analytical pharmacologist, a clinician and a statistician. The current members of the committee are noted on the QA/QC programme page of the WWARN website [1]. All practices and procedures of the WWARN QA/QC unit are documented in their own quality management system (QMS). Selected procedures will be available through the WWARN web site as a guide to participating laboratories [1]. The WWARN QA/QC unit will keep participants informed about the efficiency of the programme as a whole, any changes that are being introduced, and how any problems have been dealt with. The operation of the programme will be reviewed periodically by an independent accreditation body as part of the WWARN QA/QC units' proficiency testing provider accreditation. Overall direction of the programme will be overseen by the programme advisory committee. The unit makes use of *International Harmonized Protocol for the Proficiency Testing of (Chemical) Analytical Laboratories *as published in an IUPAC technical report [13].

Participation in the PT programme is open to all laboratories. The QA/QC unit will provide registration documents upon request. The principal investigator of a laboratory must complete the registration form and return it to the QA/QC unit who will then evaluate the application. The proficiency testing programme is limited to laboratories doing either clinical studies or research on anti-malarial drugs. In the first six months of operation, eight analytical laboratories registered with the WWARN PT programme.

PT samples will be distributed to the participants once per year. All samples will be sent in a single shipment but testing will be assessed in three cycles per year. The first cycle starts in January and ends by 30^th ^April, the second cycle starts in May and ends by 31^st ^August and the last and third cycle starts in September and ends by 31^st ^December. Samples may be requested from the WWARN QA/QC unit at any time within a cycle. However, results must be reported within two weeks after the end of the cycle. The assigned value (i.e. concentration) for each anti-malarial drug substance in each sample will be disclosed to participants together with anonymized results from all participating laboratories after each cycle. Participants should use their preferred analytical method i.e. those used by the participating laboratory for routine analysis and not versions of the method specially adapted for the proficiency test. Each laboratory will be given a unique code, and will only be able to identify their laboratory in the result reports. The results will be subjected to statistical analysis and converted into scores by the WWARN QA/QC unit. All participants will be kept fully informed of the progress of the programme at all times.

The WWARN QA/QC unit will prepare all PT samples in-house using certified reference material and characterized healthy volunteer sodium heparin plasma. Other matrixes will be added in the future. PT samples are spiked with anti-malarial drugs and drug metabolites according to valid internal SOPs. Samples are frozen at -80°C immediately after preparation. The proficiency testing programme makes use of an international courier service with experience in shipping biological material. No materials will be shipped without the required documents in place. The PT programme will initially include PT samples for dihydroartemisinin, chloroquine and lumefantrine but other drugs will be included as the programme progresses after discussions with the advisory committee.

Participating laboratories should report results in a spreadsheet format indicating the units of measurement. Results below the lower limit of quantification (LLOQ) for a specific method in a laboratory should not be reported but the LLOQ for each analyte (drug/metabolite) must be reported so that the standard deviation for proficiency assessment (SDPA) can be calculated on an individual basis for each PT sample. Results submitted after the deadline for that cycle will be rejected, and submitted results cannot be corrected or withdrawn. The reason for this stringent approach is that proficiency testing is intended to test every aspect of the analytical process, including calculating, checking, and reporting a result.

### Assessment of performance

For each drug in a cycle, a criterion of performance is determined against which the performance level obtained by a laboratory can be assessed. The performance criterion is set so as to ensure that the analytical data routinely produced by the laboratory is of a quality that is adequate for its intended purpose. The WWARN QA/QC unit continuously evaluates different approaches for assessment of performance. The goal is to select the approach which best evaluates whether the methods are fit for purpose (i.e. to quantify sample concentrations in line with international guidelines). The standard assessment below will always be carried out. The WWARN QA/QC unit will notify all participating laboratories of any change in the assessment of performance prior to each cycle. Laboratories are assessed on the difference between their result and the assigned value for each PT sample. A performance score for each PT sample will be calculated for each laboratory using the following formula:

$$z=(x-x_{\text{a}})/\text{S}\text{D}\text{P}\text{A}$$

where ***z ***is the performance score

***x ***is the participant result

***x*_a _**is the assigned value

**SDPA **is the standard deviation for proficiency assessment

### Assigned value (x_a_)

The assigned value ***x*_a _**is determined by one of the following methods:

1. formulation (i.e., the nominal value assigned on the basis of proportions of drug reference material used in spiked PT samples)

2. a consensus value (i.e., an average value derived directly from the reported results of all participating laboratories)

The number of laboratories taking part in the WWARN QA/QC programme determines what method will be used to determine the assigned value. The QA/QC unit will compare the average value of all participating laboratories with the formulation value after each cycle. If large unexplained discrepancies are found, the QA/QC unit will initiate an investigation for the root cause.

### Standard deviation for proficiency assessment (SDPA)

The revised Harmonized Protocol (HP) describes several ways in which the SDPA can be obtained. It can, for example, be determined in a proficiency test as the standard deviation of all the laboratory results (excluding significant outliers). However the HP recommends that the SDPA is a set value, which corresponds to the precision needed to perform a certain task. The HP calls this SDPA the "*fitness-for-purpose *based standard deviation for proficiency assessment [14].

The WWARN QA/QC unit will calculate the SDPA for each PT sample based on the LLOQ reported by the laboratory. This means that the same PT sample could produce different SDPA for different laboratories depending on the LLOQ for their respective method. See example below (Table 1).

**Table 1:** Example of SDPA for different LLOQ's

QC Sample	QC1		QC2		QC3		QC4	
Assigned value	5		20		100		500	
	SDPA (%)	SDPA (value)	SDPA (%)	SDPA (value)	SDPA (%)	SDPA (value)	SDPA (%)	SDPA (value)
**Lab 1 LLOQ 1**	20%	1	15%	3	15%	15	15%	75
**Lab 2 LLOQ 20**	<LLOQ		25%	5	20%	20	15%	75

Calculation of SDPA for each PT sample [15].

1 - 3×  LLOQ  ±25% of the assigned value

>3 - 10× LLOQ  ±20% of the assigned value

>10×  LLOQ  ±15% of the assigned value

### The z-score

The z-score is an indication of the proficiency of a laboratory and will be graded to the following schedule for each individual PT sample [16]:

1. |*z*| < 2: the result is considered satisfactory.

2. 2 < |*z*| < 3: the result is considered questionable.

3. |*z*| > 3: the result is considered unsatisfactory.

The WWARN QA/QC unit will provide a performance report to each participant for each cycle where the overall performance will be appraised as follows [17]:

1. **Satisfactory performance **is the successful analysis of a drug by the laboratory (More than 80% of all z-scores for a drug < 2). No further action is necessary and the laboratory demonstrated proficiency in carrying out analysis for that particular drug.

2. **Questionable performance **is when the laboratory produces more than 20% of all z-scores for a drug > 2 but less than 10% > 3. The laboratory is advised to completely investigate every aspect of the questionable and unsatisfactory results (|*z*| > 2) to prevent recurrence of this problem or any other problem identified during the investigation.

3. **Unsatisfactory performance **is when the laboratory produces more than 20% of all z-scores for a drug > 3. The laboratory is advised to completely investigate every aspect of the unsatisfactory results to prevent recurrence of this problem or any other problem identified during the investigation.

4. **Unsuccessful performance **is when the laboratory displays **unsatisfactory performance **for a drug for two consecutive or two out of three cycles. The laboratory is advised to completely investigate every aspect of the analytical methodology and re-confirm method validation performance.

5. **Critical performance **is when the laboratory displays **unsatisfactory performance **for a drug for three consecutive cycles. The laboratory is advised to cease testing until all necessary actions are taken to correct performance

Laboratories that do not meet the criteria for **successful performance **for a given drug are encouraged to contact the WWARN QA/QC unit. The QA/QC unit will provide assistance and advice to help laboratories solve quality or methodological issues that might arise during testing. Reports issued to participants will be clear and comprehensive and show the distribution of anonymized results from all laboratories together with the participant's performance score. Test results used by the WWARN QA/QC unit in all calculations will be provided, to enable participants to check that their data have been correctly entered. Reports will be made available as quickly as possible after the return of results to the WWARN QA/QC unit. Participants will receive reports in clear and simple format with summary results in graphical form (e.g., as a histogram, bar chart, or other distribution plot) with appropriate summary statistics.

Summary statistics of the programme will be posted by means of anonymous identifiers via the WWARN website. Participants will be identified in reports by code only. Participants in the proficiency testing programme may communicate their own results, including the regular programme reports, privately to a laboratory accreditation or other assessment body when required for the purpose of assessment, or to clients for the purpose of demonstrating analytical capability.

On joining the programme, participants are provided with detailed information, including:

• the range of tests available and the tests the participant has elected to undertake;

• the method of setting performance criteria;

• performance criteria applicable at the time of joining, unless criteria are set separately for each test material;

• the method(s) of determining assigned values, including measurement methods where relevant;

• a summary of the statistical procedures used to obtain participant scores;

• information on interpreting scores;

• conditions pertaining to participation (e.g., avoidance of collusion with other participants);

• the composition and method of selection of the advisory committee; and

• contact details for the WWARN QA/QC unit and any other relevant organizations.

Participants will be advised of any forthcoming changes in programme design or operation, and will be encouraged to provide feedback on all aspects of programme operation and on problems with individual test materials. In this way, participants will contribute to the development of the programme and to alerting the WWARN QA/QC unit to any unanticipated difficulty with its system and PT samples.

## Conclusion

The WWARN QA/QC programme is a dynamic programme for the distribution of certified reference standards and proficiency testing samples for anti-malarial drug measurement. It will facilitate clinical pharmacokinetic and in vitro sensitivity studies around the world. The proficiency testing programme is designed as a cooperative effort to help participating laboratories assess their ability to carry out drug analysis, resolve any potential problem areas and to improve their results - and, in so doing, to improve the quality of anti-malarial pharmacokinetic data published and shared with WWARN. To ensure the quality of pharmacokinetic data, it is important that laboratories use validated and accurate methods, but equally important that results are compared with other laboratories. By utilizing the same source of standards for all laboratories, it is possible to minimize bias arising from poor quality reference standards. By providing anti-malarial drug standards from a central point, it is possible to lower the cost of these standards. This process both assesses and empowers. Laboratories which can provide evidence of the accuracy of their analytical methods can prove to their host institutions, sponsors, and professional colleagues that they are performing at a high standard. This innovative approach improves trust, and could help translate evidence into policy and practice. It is hoped that similar programmes can be developed for other anti-infective drug measurement in tropical countries. The WWARN QA/QC unit will review the outcomes of every cycle of the programme, noting, for example, any strengths, weaknesses, specific problems, and opportunities for improvement. Every aspect of the operation of the programme, including any issues identified by the participants is continuously monitored and feedback from participants is encouraged.

This programme will hopefully facilitate for groups to conduct and analyse clinical pharmacokinetic studies of the highest quality and thereby generate desperately needed data on anti-malarial pharmacology and its impact on treatment response. The WWARN QA/QC programme is dedicated to provide a service of the highest standard and to this effect is pursuing proficiency testing provider accreditation from a nationally recognized accreditation body. It is envisaged that accreditation will be obtained as soon as the first cycle of the quality control programme is completed. Participating laboratories will then also be able to use their performance in the WWARN QA/QC programme as additional proof of their standard of service [18]. They may also quote their results in scientific publications. Contact information for the WWARN QA/QC unit can be found through the WWARN website where also registration forms for the proficiency testing programme and/or the reference material programme can be requested [1].

## Competing interests

The authors declare that they have no competing interests.

## Authors' contributions

WMW, KIB, CHS, PJG, NJW and NL developed the concept idea of a WWARN quality control programme.

NL and CL have designed the programme and developed the quality management system. CL has been responsible for the day-to-day running of the programme. CL and NL drafted the manuscript. All authors helped in manuscript preparation, read and approved the final document.
